# AI-enabled protein design facilitates future plant research and crop breeding

**DOI:** 10.1093/plphys/kiag147

**Published:** 2026-03-18

**Authors:** Yuxuan Lou, Tianhao Wu, Fan Xia, Anwen Zhao, Xiangfeng Wang

**Affiliations:** State Key Laboratory of Maize Bio-breeding, Frontiers Science Center for Molecular Design Breeding, College of Agronomy and Biotechnology, China Agricultural University, Beijing 100094, China; State Key Laboratory of Maize Bio-breeding, Frontiers Science Center for Molecular Design Breeding, College of Agronomy and Biotechnology, China Agricultural University, Beijing 100094, China; State Key Laboratory of Maize Bio-breeding, Frontiers Science Center for Molecular Design Breeding, College of Agronomy and Biotechnology, China Agricultural University, Beijing 100094, China; State Key Laboratory of Maize Bio-breeding, Frontiers Science Center for Molecular Design Breeding, College of Agronomy and Biotechnology, China Agricultural University, Beijing 100094, China; State Key Laboratory of Maize Bio-breeding, Frontiers Science Center for Molecular Design Breeding, College of Agronomy and Biotechnology, China Agricultural University, Beijing 100094, China

## Abstract

Artificial intelligence (AI) is poised to reshape the research paradigm of the life sciences by rapidly advancing the adoption of protein language models and their derivative tools. These technologies are increasingly being applied to protein structure prediction, function analysis, and protein design throughout the life sciences, and have only recently begun to gain attention within the plant science community. Moreover, while the era of AI-driven bio-breeding is on the horizon, it remains largely in the proof-of-concept stage. Therefore, there is a pressing need not only to outline the fundamental principles, models, and tools in this rapidly evolving field, but also to explore their potential applications in plant research and crop breeding. This review begins by introducing general principles and widely used models for protein understanding and generation, supported by illustrative case studies that highlight how these tools are advancing fundamental plant research. For instance, the analyses of 2 maize (*Zea mays*) genes demonstrate how a structure-aware interpretation of the relationships between mutations and protein function enables more precise hypothesis generation and facilitates experimental validation. Subsequently, the review presents generic AI-enabled protein engineering strategies and pipelines, including rational, semi-rational, refactoring, and de novo design, tailored to diverse protein engineering objectives. These approaches aim to create artificial variants and synthetic proteins with improved or novel functions to foster innovation in crop breeding. Finally, the significant challenges of applying protein design in plants are discussed, particularly in light of the limited availability of experimentally resolved protein structures and the inherent complexity of plant biological systems.

## Introduction

The 2024 Nobel Prize in Chemistry was awarded to David Baker, Demis Hassabis, and John M. Jumper for their groundbreaking contributions to protein design and structure prediction with deep learning (DL). This recognition affirms protein engineering and structural modeling as central pillars of the life sciences and highlights the broader rise of artificial intelligence (AI) for science (AI4S), which is reshaping both research and biotechnology (Cheng and Wang 2024; Li et al. 2025). In biomedicine, AI-driven methods are already accelerating the design of therapeutic proteins, antibodies, vaccines, programmable nanomaterials, and biosensors (Koh et al. 2025; Villanueva-Flores et al. 2025). Tools such as AlphaFold2 (AF2), Rosetta, RFdiffusion, and ProteinMPNN have become indispensable for computational drug design and screening, cutting development timelines from years to weeks (Leman et al. 2020; Jumper et al. 2021; Dauparas et al. 2022; Watson et al. 2023). Protein design is poised to play a similar role in plant research and breeding. Protein language models (PLMs) are DL architectures pre-trained on large protein datasets to learn universal representations for downstream protein understanding and generation tasks. With the assistance of PLMs, researchers can uncover causal genetic variations and clarify their roles in agronomic traits, and then design artificial variants or create synthetic proteins absent from nature. This approach not only accelerates trait improvement but also extends breeding beyond the limits of natural variation. The field is shifting from discovering and understanding traits to designing and synthesizing them.

Despite success in human health, protein design has only recently gained traction in plant science. Researchers must now clarify its potential, address limitations, and chart practical pathways for application. In this article, we review the principles, models, and tools driving protein design and highlight its promise in plant research and breeding. To illustrate its potential, we present 2 hypothetical maize case studies: ZmGID1 and ZmGA20ox3, proteins involved in gibberellin (GA) signaling and biosynthesis (Sun 2011; Paciorek et al. 2022). These examples show how PLMs and protein design can support hypothesis generation, mechanistic validation, and trait improvement, such as modifying GA-regulated plant height.

## Principles and tools in protein understanding tasks

Protein design starts with a deep understanding of a target protein's structure, properties, and functions before generating new sequences. The workflow is usually divided into 2 closely interrelated parts: protein understanding and protein generation. While protein understanding refers to the systematic analysis of proteins to inform engineering or biological research, protein generation involves the creation of novel protein sequences and/or structures, either by modifying existing proteins or designing new ones from scratch. Typically, understanding tasks start with protein representation learning, a process of mathematically encoding proteins into continuous vector spaces to capture biological and physicochemical properties for downstream analysis, followed by predictions of structure, properties, interactions, and mutational effects (MEs). Accurate understanding subsequently enables precise design and computational screening under defined functional or structural constraints. This section outlines the key principles and commonly used tools in protein understanding.

### Protein representation learning

Before neural networks entered structural biology, research relied on hand-crafted features to represent proteins (Chen and Li 2013; Saravanan and Gautham 2015). The arrival of DL and natural language processing transformed the field. For example, Long Short-Term Memory and Transformer models based on self-attention mechanisms, treat DNA and proteins as a “language of life,” mapping nucleotide and amino acid sequences into continuous vector spaces (Rao et al. 2019). Geometric models extend this approach to capture 3-dimensional (3D) structural patterns, using graph-based neural networks that incorporate 3D coordinates and geometric relationships to learn spatial structural patterns (Gainza et al. 2020). These representations underpin downstream tasks such as predicting protein–protein interactions (PPI) and protein–small molecule interactions (PMI). Because public datasets now contain hundreds of millions of protein sequences, PLMs have been pretrained on large-scale unlabeled sequences and structural data, along with prior biological knowledge. Such models distill universal semantics that generalize across diverse species and protein families. To leverage PLMs, researchers adopt the pretrain–finetune paradigm: a 2-stage strategy that first pretrains models on large-scale unlabeled data and then finetunes them on task-specific labeled data to optimize performance (Schmirler et al. 2024). Scaling laws describe empirical relationships showing that model performance improves predictably with increases in data size, parameter count, and computational resources. These laws have motivated the development of progressively larger PLMs (Lin et al. 2023).

PLMs generally fall into 3 categories based on the training inputs (Table 2). Sequence-only models, such as ProtTrans, ESM2, and DPLM, rely solely on residue sequences and learn contextual embeddings that are dense, low-dimensional vectors generated by neural networks to represent discrete entities, through strategies including masked language modeling (MLM), autoregressive modeling, and diffusion modeling approaches (Meier et al. 2021; Rives et al. 2021; Elnaggar et al. 2022; Lin et al. 2023; Wang et al. 2024a). While MLM is a self-supervised learning strategy in which random tokens are masked and the model predicts them, autoregressive modeling is an alternative modeling strategy where the system iteratively predicts the next element using previously generated information. In contrast, diffusion modeling is a probabilistic generative framework that produces data by learning to reverse an iterative noising process. Structure-only models, exemplified by GearNet, use geometric graphs of protein structures as input and map spatial conformations into mathematical representations, through self-prediction and contrastive learning (Zhang et al. 2022c) (Box 1). Multimodal models, including SaProt, ESM-GearNet, DPLM-2, S-PLM, OntoProtein, and ProtST, integrate information from sequences, structures, and biological annotations such as InterPro to overcome the limitations of single-source approaches (Zhang et al. 2022b; Su et al. 2023; Xu et al. 2023; Zhang et al. 2023; Wang et al. 2024b; Hayes et al. 2025; Wang et al. 2025a).

Box 1. Self-prediction and contrastive learning as pretraining tasks Self-prediction:

Self-prediction enables models to learn from unlabeled protein data by predicting masked components based on the remaining context (Zhang et al. 2022c). For residue type prediction (a core self-prediction task for protein structure modeling), let G=(V,E,R) denote a protein residue graph, where V is the set of nodes (each representing an alpha carbon of a residue), E is the set of edges, and R is the set of edge types. Let $f_{i}\in {\{\;0,1\}\;}^{21}$ represent the one-hot encoded residue type feature for node i∈V. Let M⊂V be the set of masked nodes and G∖M be the graph with residue type features of nodes in M hidden. With model parameters θ, the loss is

$$L=-\underset{i\in M}{\sum }\;\log\;p_{\theta }(f_{i}|G\setminus M)$$

Contrastive learning:

Contrastive learning aims to learn representations in which matched views are close in embedding space, while mismatched views are well separated (Zhang et al. 2022c). Let z be an embedding of one view (eg, a protein substructure view generated by subsequence cropping or subspace cropping), $z^{+\;}$ the embedding of its matched view (eg another substructure view of the same protein), and $z_{k}^{\text{-}}$ the embedding of mismatched views indexed by k (eg, substructure views from other proteins). Let S(a,b) denote a similarity function (often cosine similarity), and τ>0 a temperature scalar. A common loss is

$$L=-\log\frac{\exp\;(s(z,z^{+})/\tau )}{\exp\;(s(z,z^{+})/\tau )+\sum_{k}\;\exp\;(s(z,z_{k}^{-})/\tau )}$$

### Protein structure prediction

Accurate structures provide the foundation for protein engineering, yet experimentally resolved protein structures and complexes remain limited. DL methods overcome this gap by learning folding patterns from large-scale structural data, offering faster and more precise predictions. Among available tools for monomer structure prediction, AF2 remains the most widely recognized. The Evoformer module in AF2 utilizes attention networks to dynamically weigh the importance of input elements, which may efficiently extract conserved information embedded in multiple sequence alignments (MSAs) (Jumper et al. 2021). Despite its accuracy, MSA searches are computationally costly and time-consuming. To bypass this, ESMFold uses the ESM-2 language model to implicitly capture residue relationships and structural features without the need for MSA search (Lin et al. 2023).

The latest AF3 framework introduces a diffusion-based architecture that enables rapid and accurate modeling of complex structural assemblies, including proteins, peptides, nucleic acids, ligands, metal ions, and post-translationally modified residues (Abramson et al. 2024). Other open-source tools, such as Chai-1, Boltz-1, HelixFold3, and Protenix, replicate most AF3 functionalities, giving researchers the flexibility to adapt and deploy AF3-like models for diverse downstream applications (Boitreaud et al. 2024; Liu et al. 2024a; Wohlwend et al. 2024; Chen et al. 2025b). To systematically evaluate the performance of these all-atom prediction methods, the FoldBench benchmark was recently developed (Xu et al. 2025) (Table 1). This benchmark reveals that AF3 consistently outperforms other methods in overall accuracy for both monomeric structures and macromolecular complexes. Nevertheless, antibody–antigen complex prediction remains particularly challenging, with all evaluated methods achieving substantially lower success rates (∼20% to 50%). In protein-ligand docking tasks, current models show limited generalization to novel or dissimilar ligands, suggesting a strong dependence on the binding patterns encountered during training. Collectively, these findings suggest that AF3-like models are powerful tools for generating hypotheses, but their reliability must be carefully evaluated in a task-specific and data-dependent manner (Xu et al. 2025).

**Table 1:** Comparison of AF3-like protein structure prediction models on FoldBench.

Attribute	AF3	Boltz-1	Chai-1	HelixFold3	Protenix
Training data cutoff date (from Protein Data Bank)	2021/9/30	2021/9/30	2021/1/12	2021/9/30	2021/9/30
Protein monomer TM-score (mean)	**0**.**94**	0.94	0.93	0.94	0.93
Protein-protein DockQ success rate (%)	**72**.**93**	68.25	68.53	66.27	68.18
Protein-ligand Docking success rate (%)	**64**.**9**	55.04	51.23	51.82	50.7
Antibody-antigen DockQ success rate (%)	**47**.**9**	33.54	23.64	28.4	34.13
Protein-peptide DockQ success rate (%)	82.98	79.17	66.67	**89**.**47**	77.08
Protein-DNA DockQ success rate (%)	**79**.**18**	70.97	69.97	50.00	68.39
Protein-RNA DockQ success rate (%)	**62**.**32**	56.90	50.91	48.28	44.78
Academic use	Free	Free	Free	Free	Free
Commercial use	**Prohibited**	Free	Free	**Paid**	Free

Success rate is defined as the percentage of predictions with ligand RMSD (LRMSD) below 2 Å and protein-ligand interface LDDT (LDDT-PLI) greater than 0.8 for protein-ligand complexes, and as the percentage of predictions achieving a DockQ score of 0.23 or higher for all other interface types. The bold values represent the best values among all tools.

While AF3 does not currently account for conformational dynamics, several new tools attempt to fill this gap. AlphaFlow and BioEmu employ flow-matching strategies to learn continuous vector fields that map noise to samples from target data distributions. In contrast, ESMdiff finetunes ESM3 with masked diffusion, a variant of diffusion modeling that incorporates masking strategies (Box 2). These methods enable structural dynamics prediction at much lower cost than traditional simulation-based methods (Jing et al. 2024; Lu et al. 2024; Lewis et al. 2025).

Box 2. Diffusion and flow matching models in protein design Diffusion model

Diffusion model is a type of generative deep learning framework that creates new data (eg protein structures or sequences) by learning to reverse a gradual noising (diffusion) process (Watson et al. 2023). Starting from data samples, noise is iteratively added until only random noise remains. The model is then trained to iteratively remove this noise, step by step, thereby reconstructing realistic samples. This allows efficient sampling of complex, high-dimensional biological data.

DDPM

DDPM is a widely used diffusion model formalized as follows (Watson et al. 2023). Let $x_{0}$ represent a clean data point (such as a protein backbone structure), and $x_{t}$ be the progressively noised version at timestep t∈{1,…,T}. The forward diffusion process adds Gaussian noise:

$$q(x_{t}|x_{t-1})=N\left(\sqrt{1-\beta_{t}}x_{t-1},\beta_{t}I\right),$$

Where $q(x_{t}|x_{t\text{-}1})$ is the conditional probability distribution of $X_{t}$, N(μ,Σ) denotes a normal distribution with mean μ and covariance Σ, $\beta_{t}\in \left(0,1\right)$ is the noise level at step t, and I is the identity matrix. The goal of DDPM is to learn the reverse process $p_{\theta }(x_{t\text{-}1}|x_{t})$, parameterized by θ, which progressively denoises $x_{t}$ to recover $x_{0}$. During training, the model minimizes the difference between the predicted and true noise, enabling the generation of novel protein structures starting from pure noise.

Masked diffusion:

Masked diffusion is a variant of diffusion-based generative modeling in which corruption is performed by masking rather than adding Gaussian noise. This approach is particularly well suited for discrete data, such as protein sequences, where masking provides a more natural and semantically meaningful form of perturbation (Lu et al. 2024).

Flow matching:

Flow matching is an alternative generative modeling approach that learns a continuous vector field mapping noisy samples to clean data in a single step, thereby bypassing the need for iterative denoising. Mathematically, it defines an ordinary differential equation (ODE):

$$\frac{dx_{t}}{dt}=v_{t}(x_{t})$$

Where $x_{t}$ flows smoothly from noise to data. Training optimizes $v_{t}$ to match the probability flow of the data distribution (Bose et al. 2024; Huguet et al. 2024).

### Protein property prediction

Predicting protein properties represents another critical component of protein understanding, as it provides constraints for designing and screening proteins with desired functions. Recent DL-based methods leverage advanced sequence and residue representations to significantly improve both accuracy and efficiency compared with earlier physicochemical-based approaches (Table 2). In enzyme design, TemStaPro, UniKP, and EpHod, all developed from pre-trained PLMs, allow direct prediction of thermal stability, enzyme kinetics, and optimal pH conditions from amino acid sequences (Yu et al. 2023a; Pudžiuvelytė et al. 2024; Gado et al. 2025). UniKP further integrates substrate information encoded in SMILES (Simplified Molecular Input Line Entry System) strings, combining chemical notation with protein representations to predict kinetic constants through regression models. Other important design-related properties, such as solubility and expression abundance, can now be estimated using tools like NetSolP and MPB-EXP (Thumuluri et al. 2022; Liu et al. 2025b).

**Table 2:** Protein language models and derivative tools.

Category	Model name	Derivative tools or models	References
Sequence-only	ProtTrans	TemStaPro, UniKP, CataPro, ProT-Diff, GPsite	Elnaggar et al. (2022)
	MP-TRANS	MPB-EXP	Liu et al. (2025)
	ESM-1b	NetSolP, CLEAN	Rives et al. (2021)
	ESM-1v	EpHod	Meier et al. (2021)
	ESM2	ESMfold, PepMLM, surfdock, EVOLVOpro	Lin et al. (2023)
	DPLM		Wang et al. (2024a)
Structure-only	GearNet	GearBind	Zhang et al. (2022c)
Multimodel	SaProt	SSAlign, SaProt-EVE	Su et al. (2023)
	ESM-GearNet		Zhang et al. (2023)
	DPLM-2	DPLM-2-Bit	Wang et al. (2024b)
	S-PLM		Wang et al. (2025a)
	OntoProtein		Zhang et al. (2022b)
	ProtST	PROTLLM	Xu et al. (2023)
	ESM3	ESMdiff, EiRA	Hayes et al. (2025)

### Protein interaction prediction

Identifying hotspot residues at PPI or PMI sites provides crucial guidance for protein design tasks, especially in binder, antibody, and nanobody engineering. Some design tools rely on user-specified hotspots, which anchor binders to predefined interfacial regions. Widely used tools for interaction prediction include MaSIF, GPSite, and SurfDock (Table 3). MaSIF segments the molecular surface into overlapping geodesic patches and applies geometric DL to generate interaction fingerprints that capture key binding patterns (Gainza et al. 2020). GPSite constructs residue-level graphs enriched with geometric features and uses graph neural networks (GNNs) to predict binding residues for diverse biomolecules (Yuan et al. 2024). Both tools help pinpoint candidate hotspot residues, improving the precision and efficiency of binder design. In contrast, SurfDock focuses on pocket-centric protein–ligand docking by integrating sequence, structural, and surface information within a diffusion-based generative framework to predict ligand binding poses (Cao et al. 2025).

**Table 3:** Protein design models and generic tools.

Name	Application	Reference or link	AI-based
PLIP	PPI and PMI analysis	Salentin et al. (2015)	No
PDBePISA	PPI and PMI analysis	Krissinel and Henrick (2007)	No
Surf2Spot	Protein binding sites prediction	https://github.com/AnwZhao/Surf2Spot	Yes
MaSIF	Protein binding sites prediction	Gainza et al. (2020)	Yes
GPsite	Protein binding sites prediction	Yuan et al. (2024)	Yes
MVGNN-PPIS	Protein binding sites prediction	Meng et al. (2025)	Yes
Chainsaw	Protein domain splitting	Wells et al. (2024)	Yes
RFdiffusion	Backbone generation	Watson et al. (2023)	Yes
RFdiffusion-All-Atom	Backbone generation	Krishna et al. (2024)	Yes
ProteinMPNN	Inverse folding	Dauparas et al. (2022)	Yes
ESM-IF1	Inverse folding	Hsu et al. (2022)	Yes
LigandMPNN	Inverse folding	Dauparas et al. (2025)	Yes
RFdiffusion2	De novo enzyme backbone design	Ahern et al. (2026)	Yes
ProT-Diff	Antimicrobial peptide design	Wang et al. (2024c)	Yes
PepMLM	Peptide binder design	Chen et al. (2025a)	Yes
BindCraft	Binder design	Pacesa et al. (2025)	Yes
FoldCraft	Binder/antibody design	Rustamov and Baev (2025)	Yes
IgGM	Antibody design	Wang et al. (2025c)	Yes
AiCE	Protein optimization	Fei et al. (2025)	Yes
EVOLVEpro	Iterative protein optimization	Jiang et al. (2025)	Yes
FireProt ASR	Ancestral sequence reconstruction	Khan et al. (2021)	No
SaProt	Protein ME prediction	Su et al. (2023)	Yes
ProtREM	Protein ME prediction	Tan et al. (2025)	Yes
PoET	Protein ME prediction	Truong and Bepler (2023)	Yes
VespaG	Protein ME prediction	Marquet et al. (2024)	Yes
structural-evolution	Binding affinity changes prediction	Shanker et al. (2024)	Yes
Flex ddG	Binding affinity changes prediction	Barlow et al. (2018)	No
AlphaFold2	Protein structure prediction	Jumper et al. (2021)	Yes
RoseTTAFold	Protein structure prediction	Baek et al. (2021)	Yes
ESMfold	Protein structure prediction	Lin et al. (2023)	Yes
AlphaFold-Multitimer	Protein complex structure prediction	Evans et al. (2021)	Yes
AlphaFold3	Biomolecular structure prediction	Abramson et al. (2024)	Yes
RoseTTAFold-All-Atom	Biomolecular structure prediction	Krishna et al. (2024)	Yes
Chai-1	Biomolecular structure prediction	Boitreaud et al. (2024)	Yes
P2PXML	Antigen-antibody affinity prediction	Bandara et al. (2025)	Yes
UniKP	Enzyme activity prediction	Yu et al. (2023a)	Yes
CataPro	Enzyme activity prediction	Wang et al. (2025d)	Yes
TemStaPro	Protein thermal stability prediction	Pudžiuvelytė et al. (2024)	Yes
NetSolP	Protein solubility prediction	Thumuluri et al. (2022)	Yes
MPB-EXP	Protein abundance prediction	Liu et al. (2025)	Yes
EpHod	Enzyme optimal pH prediction	Gado et al. (2025)	Yes
SurfDock	Protein-ligand docking	Cao et al. (2025)	Yes
GALigandDock	Protein-ligand docking	Leman et al. (2020)	Yes
RosettaDock	Protein-protein docking	Lyskov and Gray (2008)	Yes
AutoDock Vina	Protein-ligand docking	Trott and Olson (2010)	Yes
Uni-Dock	Protein-ligand docking	Yu et al. (2023b)	Yes
GROMACS	Protein molecular dynamics simulation	Abraham et al. (2015)	No
Amber	Protein molecular dynamics simulation	Case et al. (2005)	No
BioEmu	Protein ensemble prediction	Lewis et al. (2025)	Yes
CodonTransformer	Codon optimization	Fallahpour et al. (2025)	Yes
TM-align	Protein structure alignment	Zhang and Skolnick (2005)	No
US-align	Biomolecular structure alignment	Zhang et al. (2022a)	No

### Mutational effect prediction

Predicting ME of non-synonymous substitutions plays a central role in rational and semi-rational protein design, since even a single amino acid residue change can alter function dramatically (Li et al. 2022; Notin et al. 2023). While rational design uses mechanistic and structural understanding to introduce targeted mutations with predictable effects, semi-rational design usually combines computational prediction and empirical screening to identify high-fitness mutations. In a semi-rational design task, zero-shot prediction estimates ME without prior experimental data, and typically predicts the fitness of residue substitutions by modeling the interplay of sequence, structure, and functional conservation captured in MSA or PLMs. SaProt improves this approach by integrating structural information into PLMs through a structure-aware vocabulary that unifies protein sequences and predicted structures, thereby increasing ME prediction accuracy (Su et al. 2023). VenusREM also ranks among the best performers in the ProteinGym benchmark, using a retrieval-enhanced protein language model (ProtREM) that leverages sequence homology to improve predictions (Tan et al. 2025). For iterative optimization in a semi-rational design task, ME prediction can be combined with few-shot active learning, a strategy where models select the most informative samples for experimental testing, optimizing performance with minimal data, as in EVOLVEpro (Jiang et al. 2025). Other approaches, such as structural-evolution and DeepDirect, focus specifically on predicting ME at residues involved in PPI (Lan et al. 2024; Shanker et al. 2024). Beyond screening mutants, these ME methods can also guide the targeted selection of sites for gene editing.

## Principles and tools in protein generation tasks

Recent advances in DL have made it possible to generate novel protein structures and sequences with both precision and efficiency. Generative models now tackle 2 main challenges: creating backbone structures and producing functional sequences. Below, we outline the key principles and methodologies/tools that drive progress in structure and sequence generation.

### Structure generation task

A protein backbone refers to the 3D arrangement of a protein's polypeptide chain, excluding side chains, and many recent tools for protein backbone design rely on diffusion-based generative models (Table 3). Among these, RFdiffusion has emerged as one of the most widely used. It combines a denoising diffusion probabilistic model (DDPM) with the sequence-to-structure prediction capacity of RoseTTAFold, which is essentially a diffusion model generating data by sequentially denoising Gaussian noise through learned reverse processes (Baek et al. 2021; Watson et al. 2023) (Box 2). In practice, RFdiffusion reuses the pre-trained RoseTTAFold network but replaces its sequence input with noisy 3D backbone coordinates and orientations, while preserving the original architecture. The weights are then fine-tuned using a diffusion-based objective that progressively denoises these geometric inputs. This process effectively transforms a structure-prediction model into a generative framework for de novo protein design.

Chroma also employs a diffusion-based framework for generating backbone structures (Ingraham et al. 2023). In contrast to RFdiffusion, it allows users to impose flexible design constraints, such as overall molecular shape, secondary structure class, or even natural-language descriptions of the intended protein, making it highly adaptable for customizable protein design. The all-atom version of RoseTTAFold and RFdiffusion (RFAA) introduced a further innovation by incorporating residue-level protein representations together with atomic graph representations of small molecules and covalent modifications (Krishna et al. 2024). This unified multi-track architecture includes chemical element types, bond connectivity, and stereochemistry, enabling simultaneous modeling of protein backbones and non-protein biomolecules. In practice, RFAA is often combined with LigandMPNN to design proteins that specifically recognize or bind small molecules (Dauparas et al. 2025).

Whereas diffusion models dominate the field, alternative approaches are emerging. Flow matching methods directly learn continuous vector fields that map random noise into structured proteins, bypassing the stochastic, stepwise denoising used in diffusion models (Box 2). This approach typically leads to faster inference with fewer sampling steps, lower computational cost, and, in many cases, comparable or improved structural quality (Bose et al. 2024; Huguet et al. 2024). These features make flow matching a promising alternative for protein design applications where efficiency is a key requirement.

### Sequence generation task

Sequence generation focuses on designing amino acid sequences that achieve desired functional objectives. Depending on the workflow, the process can be structure-independent or structure-dependent. Structure-independent generation typically uses PLMs to learn the distribution of amino acid sequences from large protein datasets and then samples new ones from this space. Models such as ProtGPT2 and ProGen exemplify this approach (Ferruz et al. 2022; Madani et al. 2023). Because no explicit structural input is required, these methods are particularly useful for designing antimicrobial peptides or peptide binders, where precise backbone scaffolds are less critical (Wang et al. 2024c; Chen et al. 2025a). In contrast, structure-dependent generation incorporates structural constraints to ensure that the designed sequences fold into a specific conformation and exhibit target activities. A representative example is the integration of RFdiffusion and ProteinMPNN, where a predefined backbone from RFdiffusion serves as the conditioning input for ProteinMPNN in a process known as inverse folding (Box 3). Previous energy-based methods, such as Rosetta Packer and ABACUS, also achieved inverse folding by minimizing energy with scoring functions (Alford et al. 2017; Xiong et al. 2020), but DL-based approaches now provide greater accuracy and speed. Tools such as ESM-IF1 and ProteinMPNN encode backbone geometry with a GNN encoder and generate sequences with an autoregressive decoder (Hsu et al. 2022). LigandMPNN further extends this architecture by incorporating small-molecule information, allowing the design of sequences adapted to interactions with ligands, nucleic acids, or metal ions.

Box 3. Inverse folding and hallucination design for protein generation Inverse folding

Inverse folding refers to the computational problem of identifying amino acid sequences ***s*** that fold into a predefined protein backbone structure ***B*** (Dauparas et al. 2022). Formally, inverse folding aims to find sequences maximizing the conditional probability:

$$s^{*}=\arg\;\underset{s}{\max}\;P(s|B)$$

Where ***B*** denotes the fixed 3D coordinates of the protein backbone (eg, positions of alpha carbons and main chain atoms), ***s*** is an amino acid sequence. The goal is to design sequences that are structurally compatible with a target fold, thereby enabling the generation of stable and potentially functional proteins.

Hallucination design

Hallucination design is a computational strategy that generates novel protein sequences and structures by iteratively optimizing a design objective through a deep learning model. In BindCraft, hallucination design uses backpropagation through a pretrained protein structure prediction network (AlphaFold-Multimer) to optimize amino acid sequences of a binder protein such that the predicted complex formed with a target protein satisfies predefined structural and interface criteria.

The core mathematical formulation involves minimizing a loss function L(s)

$$s^{*}=\arg\;\underset{s}{\min}\;L(s)=\arg\;\underset{s}{\min}\;(L_{\mathrm{conffence}}(s)+L_{\mathrm{interface}}(s,t)+L_{\mathrm{structural}}(s))$$

Where s denotes the binder sequence, t represents the fixed target protein, $L_{\mathrm{conffence}}$ measures the prediction confidence of the folded binder, $L_{\mathrm{interface}}$ encourages favorable intermolecular contacts and interface properties between binder and target, promoting strong and specific binding. $L_{\mathrm{structural}}$ imposes structural priors, such as secondary structure content (eg helicity) or compactness (eg radius of gyration). Using gradient-based optimization on sequence representations (encoded as continuous logits or probabilities), the binder sequence s is iteratively updated by backpropagating gradients of L(s) through AlphaFold-Multimer's neural network weights, effectively “hallucinating” sequences that fold into stable structures and form the desired interface with the target protein (Evans et al. 2021; Pacesa et al. 2025).

Another important structural constraint is “co-folding,” where a new sequence is optimized to interact with a given target. Hallucination design is a widely used strategy here: it iteratively refines sequences by backpropagating gradients from a structure prediction DL model, such as AlphaFold-Multimer networks, as implemented in BindCraft (Box 3). This approach enables the creation of functional binder sequences without requiring a pre-defined backbone (Evans et al. 2021; Pacesa et al. 2025). However, hallucination-based methods are computationally intensive because they must explore large sequence-structure landscapes. Efficiency can be improved by constraining the search space to specific interfacial regions or hotspot residues. Surf2Spot is one example: it predicts likely binding surfaces and anchor residues on target proteins, narrowing the search and thereby improving both efficiency and accuracy in binder design.

## Types of protein design for different goals

Protein design spans 4 main strategies: rational, semi-rational, refactoring, and de novo design, based on the goals, tools, and workflows involved. The first 3 optimize protein function using natural templates, whereas de novo design generates entirely new proteins.

### Exemplary cases of protein design in plant research

Rational design introduces point mutations at specific residues, each of which is mechanistically interpretable and rationally inferable. For example, AF2-Multimer predicted interactions between soybean pectin methylesterase (PME) inhibitor 1 (GmPMI1) and PME proteins from plants and pathogens. Interfacial residues forming strong hydrogen bonds were identified, and targeted mutations weakened GmPMI1's interaction with endogenous plant PMEs while preserving high binding affinity to pathogen PMEs, thereby selectively blocking pathogen infection (Xia et al. 2024).

Semi-rational design becomes useful when mechanistic knowledge is incomplete. Here, PLMs and DL approaches predict the effects of all possible mutations, allowing researchers to explore the mutational fitness landscape and select a focused library of mutants for experimental screening. The AiCE framework demonstrates this strategy: it uses inverse folding models such as ESM-IF1, ProteinMPNN, and LigandMPNN to nominate candidate single or combinatorial mutations in CRISPR-Cas enzymes, improving protein function while reducing the number of required experiments (Fei et al. 2025). Semi-rational design can also integrate active learning frameworks for directed evolution. For instance, EVOLVEpro iteratively fine-tunes its predictive model using limited experimental data generated in each optimization cycle until the desired protein function is achieved (Jiang et al. 2025).

Refactoring design explores broader sequence space to improve stability, solubility, expression, or partially novel functions, enabling proteins to outperform natural counterparts. A common approach is to retain key catalytic or substrate-binding residues while redesigning the surrounding sequence using inverse folding algorithms like ProteinMPNN (Sumida et al. 2024). New functions can be engineered by incorporating functional fragments from heterologous proteins. For instance, fusing a peptide to an Arabidopsis nucleotide-binding leucine-rich repeat protein allowed the remodeled receptor to detect pathogen-derived proteases, conferring broad-spectrum antiviral immunity (Wang et al. 2025b). Other examples include engineered bioPROTACs (biological Proteolysis-Targeting Chimeras) that combine binders, linkers, and E3 ubiquitin ligase components to target specific proteins for degradation (Lim et al. 2020). Ancestral sequence reconstruction is another refactoring strategy: resurrected ancestral Rubisco variants showed higher catalytic efficiency and enhanced enzymatic performance compared to modern versions (Lin et al. 2022).

*De novo* design creates proteins entirely from scratch, a process previously considered unattainable due to the vast sequence space (Bryngelson et al. 1995). Recent DL advances have dramatically increased the success rate of experimentally validated designs. A primary application is the generation of mini-protein binders that interact with target proteins, peptides, or small molecules. For example, RFdiffusion and ProteinMPNN have been used to design inhibitors capable of neutralizing lethal snake venoms (Vázquez Torres et al. 2025). In plants, de novo binder design enables the creation of binders targeting either exogenous or endogenous proteins, thereby allowing precise and programmable modulation of PPIs to engineer traits such as disease resistance, stress tolerance, and developmental regulation (Iqbal et al. 2026). Beyond binder design, de novo design approaches have also been employed to create other functional proteins. Notably, a recent study demonstrated the computational design of proteins incorporating excitonically coupled chlorophyll special pairs, enabling precise control over photophysical properties relevant to photosynthesis and energy conversion, which could potentially be adapted for optimizing photosynthetic efficiency in plants (Ennist et al. 2024). Because de novo approaches can produce vast numbers of candidate sequences, computational screening is as important as the design process itself. Structure prediction tools like AF2 assess whether designed binders adopt the intended conformation and bind to the desired surface. By leveraging computational tools, the success rate of experimental validation can increase by nearly an order of magnitude (Bennett et al. 2023). In addition to structural feasibility, binding affinity to substrates, compounds, or peptides, as well as pre-organization toward enzymatic substrates, can be estimated with biophysical or DL-based predictors (Lyskov and Gray 2008; Trott and Olson 2010; Yu et al. 2023a; Bandara et al. 2025; Lauko et al. 2025). Protein properties such as solubility, thermal stability, and expression abundance are equally important for function in cellular environments, and dedicated models can filter out poor candidates *in silico*.

### Generic tools and pipelines for protein design

**Figure 1:**
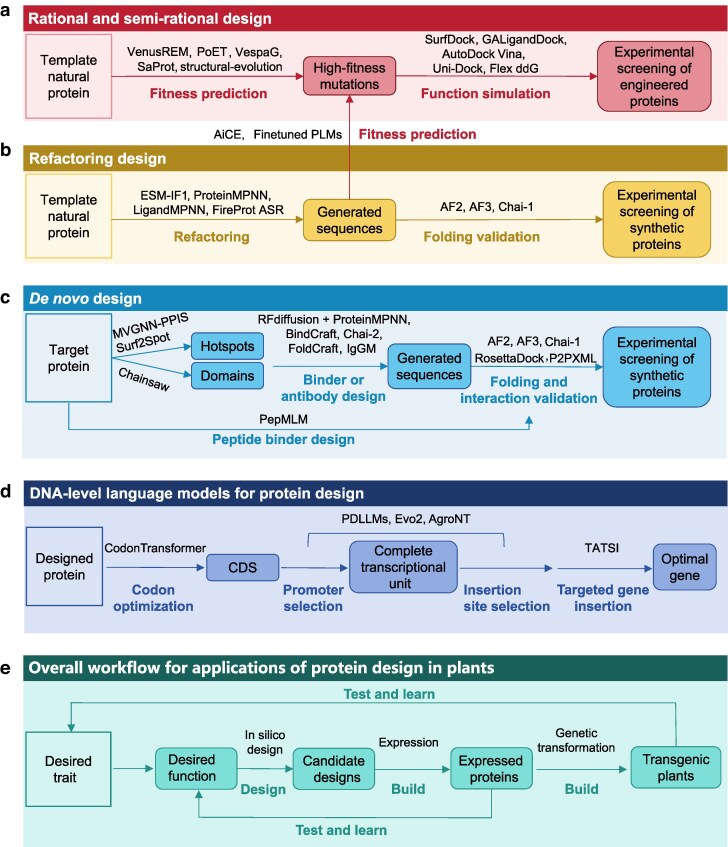
Integrated methods and workflows for protein design and plant applications. Generic tools and pipelines for protein rational and semi-rational design **(a)**, refactoring design **(b)**, and de novo design **(c)**. **(d)** DNA-level language models for protein design. **(e)** Overall workflow for applications of protein design in plants.

In this section, we integrate generic protein design strategies with complementary tools, combining structural, functional, and property-based predictions to build adaptable pipelines for diverse applications (Fig. 1). This modular framework supports efficient, precise protein engineering across diverse applications. For rational and semi-rational design, when a natural protein already fulfills basic functional requirements, ME prediction models such as SaProt, VenusREM, PoET, and VespaG identify high-fitness variants while minimizing experimental effort (Fig. 1a). Structural-evolution predicts MEs at PPI interfaces to prioritize mutations that strongly affect function, enabling targeted modulation of binding affinity. Structural modeling, combined with protein–ligand docking tools including GALigandDock, Uni-Dock, SurfDock, and AutoDock Vina, assesses whether engineered mutants preserve native substrate-binding sites, guiding the selection of promising candidates. Mutants predicted to significantly alter PPIs can be further evaluated using the more accurate Flex ddG method to improve screening precision and enhance success rates in downstream applications.

Refactoring design is to enhance native protein function or biophysical properties through the exploration of broader sequence space (Fig. 1b). Inverse folding algorithms such as ESM-IF1, ProteinMPNN, or LigandMPNN redesign sequences based on native protein structures, while ancestral sequence reconstruction tools like FireProt ASR generate variants from natural protein families. These approaches can yield proteins with improved expression, thermal stability, and functional performance, and the resulting sequences can guide subsequent ME prediction. Redesigned sequences are evaluated using structural prediction tools such as AF2, AF3, and Chai-1, with metrics including predicted TM-score, RMSD, and IPTM score to ensure proper folding and molecular co-folding.

*De novo* design of binders, antibodies, and nanobodies generally leverages target protein structural information (Fig. 1c). Binders are generated using RFdiffusion combined with sequence-design methods such as ProteinMPNN or BindCraft, focusing on predefined interaction hotspots. For large target proteins, segmentation or domain-partitioning tools (eg Chainsaw) reduce computational complexity. Hotspot residues predicted by models such as MVGNN-PPIS or Surf2Spot help concentrate design efforts, accelerating development. Designed binders undergo pre-screening with structural prediction models to verify folding and functional conformations before experimental testing. Antibody and nanobody design follows a similar workflow, which includes domain segmentation, antigen epitope site identification, sequence design (using Chai-2, FoldCraft, or IgGM), and multi-criteria filtering based on structural accuracy, predicted affinity (eg P2PXML), and binding energy estimates (eg RosettaDock). This integrated approach enhances functional efficacy and increases experimental success rates. When experimental or predicted structural data are unavailable, PepMLM can design binding peptides directly from protein sequences.

For all 3 strategies above, experimental screening is essential for selecting functional proteins from candidate designs. When the function of a target protein can be indirectly assessed through a cellular system, in vivo screening represents an efficient approach. For example, for the key photosynthetic enzyme Rubisco in plants, Rubisco-dependent microbial strains can be engineered such that microbial growth directly correlates with Rubisco activity (Gionfriddo et al. 2025; McDonald et al. 2025; Prywes et al. 2025). For binder screening, well-established in vivo validation platforms, such as luciferase complementation and yeast 2-hybrid assays, are widely employed (Fields and Song 1989; Luker et al. 2004). When protein function cannot be readily observed within cells, in vitro display technologies, including yeast surface display, bacterial surface display, phage display, and ribosome display, provide high-throughput screening alternatives that do not require protein purification (Smith 1985; Boder and Wittrup 1997; Hanes and Plückthun 1997; Ståhl and Uhlén 1997). Specifically, yeast surface display has been successfully applied to identify highly active polyethylene terephthalate-degrading enzymes (Cribari et al. 2023). In addition to display-based screening, in vitro validation platforms enable precise quantitative characterization of protein function. For instance, surface plasmon resonance assays allow accurate determination of the kinetic and thermodynamic binding parameters between designed binders and their target proteins (Capelli et al. 2023). Given that protein purification is labor-intensive and time-consuming, such quantitative validation is typically reserved for the final stage of experimental assessment.

It is important to note that protein function is ultimately encoded at the DNA level and regulated by the genomic context (Fig. 1d). For designed protein sequences, codon optimization algorithms based on pre-trained language models, such as CodonTransformer, can be employed to generate coding sequences that support efficient expression in the host species (Fallahpour et al. 2025). Furthermore, fine-tuned DNA language models, such as PDLLMs, Evo2, and AgroNT, can predict features such as promoter strength and chromatin accessibility within the host genome (Mendoza-Revilla et al. 2024; Liu et al. 2025a; Brixi et al. 2026). Predictions of promoter strength facilitate the selection or rational design of promoter elements that drive high-level expression of the designed protein, for example through genetic algorithms or related optimization strategies (Li et al. 2024). In parallel, chromatin accessibility predictions assist in identifying genomic insertion sites that are permissive to stable and functional transgene expression. Finally, targeted gene insertion technologies, such as transposase-assisted target-site integration, enable the precise integration of complete transcriptional units encoding designed proteins into optimal genomic loci of the host organism (Liu et al. 2024b).

Overall, applications of protein design in plants follow an iterative design–build–test–learn cycle (Li et al. 2025) (Fig. 1e). During the design phase, appropriate protein design algorithms are selected based on the intended application to generate candidate protein sequences. The subsequent build, test and learn phases are typically implemented at 2 levels: laboratory and field. At the laboratory stage, candidate proteins are expressed (build) and experimentally screened for the desired functions (test). Data generated from these assays can then be used to refine design targets or to fine-tune protein design algorithms (learn). At the field stage, the DNA sequences corresponding to laboratory-validated designs are introduced into the target plants via genetic transformation (build), and designs that confer the desired phenotypic traits are selected (test). Field-level performance data can further inform iterative improvements in protein design objectives and overall engineering strategies.

## Protein design reshapes the paradigm of plant research

Plant research is shifting from forward and reverse genetics to the AI4S paradigm. Traditionally, researchers mapped genes linked to phenotypes and validated their roles through transgenic overexpression, knockout, knockdown, or genome editing, often concluding with mechanistic insights from crystal structures. The AI4S framework reverses this order: hypotheses arise from protein understanding tasks supported by PLMs and other DL tools, then protein design strategies guide precise biotechnological interventions to generate materials for phenotypic validation and trait screening. As an example, we examine the well-studied gibberellin (GA)–GID1–DELLA module in Arabidopsis and rice (Shimada et al. 2008; Islam et al. 2025), showing how understanding and design tools apply to its ortholog in maize.

**Figure 2:**
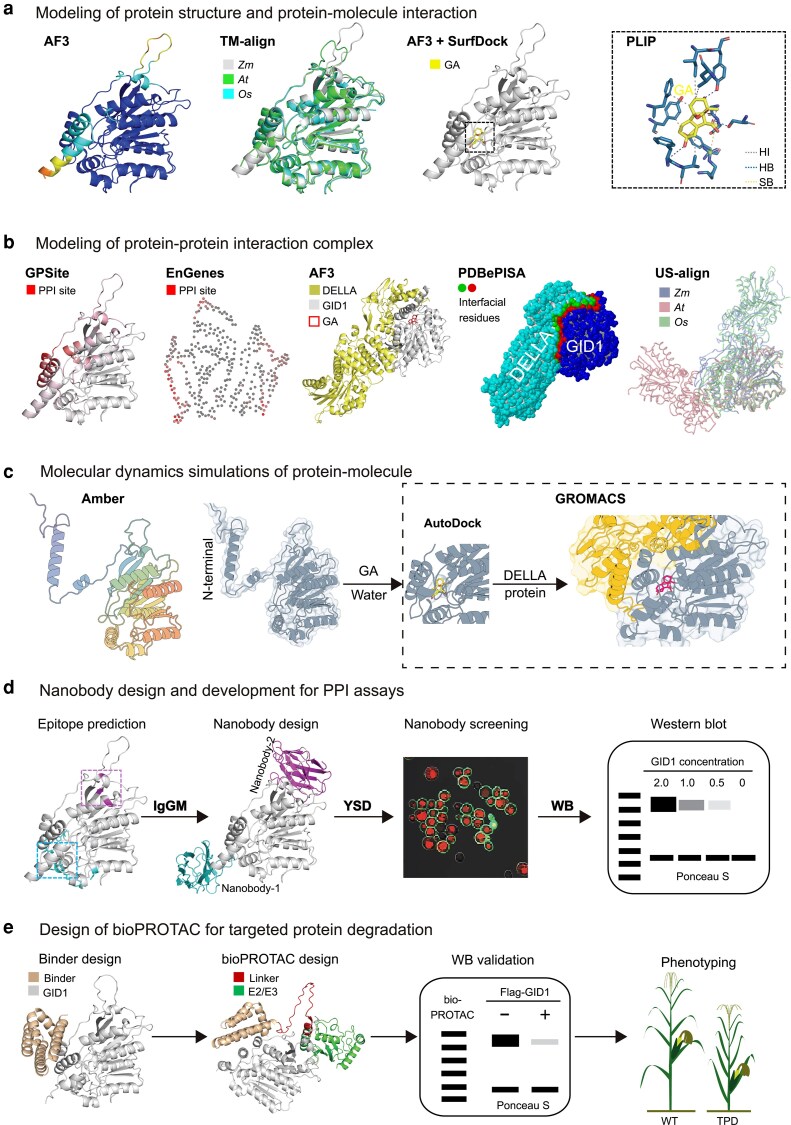
Protein design facilitates hypothesis generation and validation in plant research. **(a)** Tools for modeling the 3D structure of ZmGID1 and its interaction with gibberellin (GA). PLIP infers interaction types between ZmGID1 and GA (HI: hydrophobic interaction; HB: hydrogen bond; SB: salt bridge). **(b)** Prediction of protein–protein interaction (PPI) sites on ZmGID1, and modeling of the ZmGID1-ZmDELLA-GA complex. EnGens maps residue 3D coordinates into 2 dimensions. **(c)** Molecular dynamics simulation of the GID1-DELLA-GA complex using Amber, AutoDock, and GROMACS. Using Amber to perform molecular dynamics simulation of ZmGID1 can capture the shifts of the N-terminal helix. After docking GA into the pocket of ZmGID1 with AutoDock, molecular dynamics simulation of the ZmGID1–ZmDELLA–GA complex using GROMACS show that the complex remains stable and the N-terminal helix no longer shifts. **(d)** Pipeline for designing synthetic nanobodies to study ZmGID1. Two epitope surfaces on ZmGID1 are predicted by Surf2Spot, including hotspot residues used for nanobody design via IgGM. Nanobody screening is performed using yeast surface display (YSD) assays. ZmGID1 is tagged with EGFP, nanobodies with mCherry, and co-localization of green and red fluorescence indicates successful designs. These nanobodies can be used in molecular studies such as western blotting (WB), ELISA, co-immunoprecipitation, and subcellular localization assays. **(e)** Functional validation of ZmGID1 with bioPROTAC for targeted protein degradation (TPD). BindCraft designs binders, while the 3D structure of ZmGID1, bioPROTAC (binder-linker-E3), E2, and ubiquitin complexes can be predicted, enabling identification of effective bioPROTACs. Constructs are first validated by WB and then transgenically expressed in maize to assess phenotypic effects. The related predictive structures are generated by AF3.

We began by generating an AF3 prediction of ZmGID1, for which no experimentally resolved structure is currently available (Fig. 2a). Notably, although GID1 structures from rice and Arabidopsis deposited in the Protein Data Bank show a closed conformation of the N-terminal helical switch, the AF3 prediction exhibits a low confidence score (pLDDT) in this region, suggesting potential conformational flexibility or dynamic behavior. This observation is consistent with previously reported dynamics of the same region in Arabidopsis. Together, these findings indicate that AF3 captures intrinsic folding and dynamic properties of the protein rather than relying predominantly on homologous template structures from structural databases in contrast to traditional homology-based approaches such as SWISS-MODEL (Fig. S1). Structural alignment across Arabidopsis, rice, and maize using TM-align provides a more reliable basis for identifying functional orthologs than sequence similarity alone (Zhang and Skolnick 2005). AF3 first predicts the GA–GID1 complex structure; SurfDock then uses this model to dock GA into ZmGID1, and PLIP characterizes the interaction forces (Salentin et al. 2015). AF3 models the GA–GID1–DELLA complex, with interfacial residues identified by GPSite and PDBePISA (Krissinel and Henrick 2007), and US-align used for cross-species comparison (Fig. 2b) (Zhang et al. 2022a). Protein function often relies on dynamic conformational changes, and the GA–GID1–DELLA complex illustrates this: GA binding induces a shift in the N-terminal helical switch of GID1, creating a DELLA-binding surface, as shown in Arabidopsis. In maize, this mechanism can be reconstructed using molecular dynamics simulations (Amber and GROMACS) and AI-enhanced sampling tools such as BioEmu (Case et al. 2005; Abraham et al. 2015; Lewis et al. 2025), yielding a testable mechanistic model (Fig. 2c).

Protein design tools then aid hypothesis validation. PLM-based ME prediction highlights critical interfacial and GA-contacting residues. Single-base or prime editing at these sites tests their effect on GA signaling and phenotype. Deleting parts of the N-terminal helical switch probes whether maize mirrors the Arabidopsis mechanism. Synthetic binders or nanobodies designed to target ZmGID1 can support assays such as western blotting, co-immunoprecipitation, and subcellular localization (Fig. 2d). Validated binders and nanobodies can also be fused with linkers and engineered ubiquitin-conjugating enzymes (E2) or ubiquitin ligases (E3) to create a system for targeted ZmGID1 degradation, which can be regarded as a genetically encoded fusion protein that directs degradation of specific proteins via the ubiquitin-proteasome system (Fig. 2e, Fig. S2). Transgenic expression of this bioPROTAC construct would allow phenotypic evaluation of reduced ZmGID1 levels, complementing genome editing approaches.

## Applications of protein design in future crop breeding

**Figure 3:**
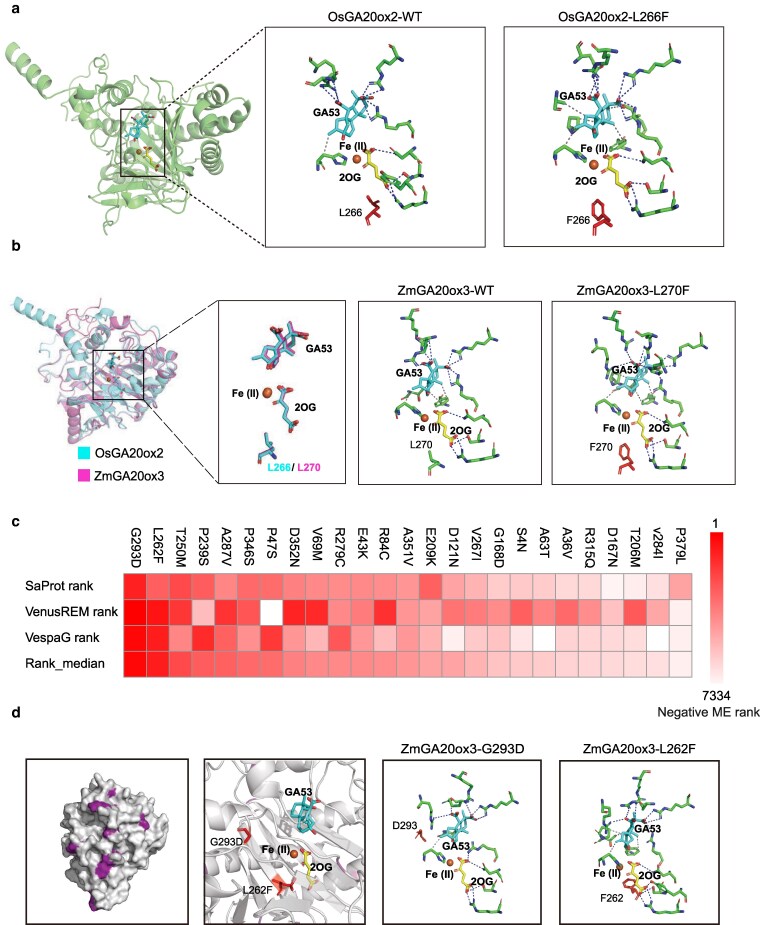
Rational and semi-rational design for plant breeding. **(a)** PLIP analysis of the interaction changes caused by the L266F mutation in OsGA20ox2 with the ligand. **(b)** TM-align analysis shows identical GA53 conformations in the pockets of ZmGA20ox3 and OsGA20ox2. PLIP analysis indicates that the maize L270F mutation induces a conformational change in GA53 similar to the rice L266F mutation. **(c)** Effects of all possible single amino acid substitutions in ZmGA20ox3 ranked by SaProt, VenusREM, and VespaG. The median values of these rankings are used as a comprehensive indicator to evaluate the MEs. **(d)** Location and impact of the predicted most deleterious G293D and L262F mutations among 26 EMS-induced nonsynonymous variants. PLIP analysis reveals that they lead to alterations in a series of bonds involved in GA53 binding. The related predictive structures are generated by Chai-1.

Breeding harnesses natural or artificial genetic variation to achieve desired traits. PLMs and their derivative tools now enable us to reveal the mechanistic basis of natural variants and engineer artificial ones, paving the way for AI-driven bio-breeding. We outline 4 hypothetical scenarios illustrating how protein design can advance plant breeding, which correspond to rational, semi-rational, refactoring, and de novo design. The first example involves ZmGA20ox3, the maize ortholog of the rice “green revolution” protein SD1 (Semi-Dwarf 1, OsGA20ox2). Substituting Leu266 with Phe (L266F) in rice causes dwarfism by disrupting GA biosynthesis (Fig. 3a) (Spielmeyer et al. 2002). Structural alignment with TM-align shows that maize L270 corresponds to rice L266; both residues sit in the pocket but do not directly contact GA53. PLIP analysis indicates that the OsL266F and ZmL270F mutations similarly perturb bonding interactions involved in the binding of GA53 and 2OG, likely through steric hindrance from the rigid benzyl side chain of Phe (Fig. 3b). This supports designing an L270F mutation in ZmGA20ox3 via prime editing (PE) or base editing (BE). We can also explore the EMS-induced non-synonymous mutations from the maize missense-effect prediction database (https://maizemep.com/). We integrated the results from the ME prediction tools SaProt, VenusREM, and VespaG to comprehensively assess the effects of all potential single amino acid substitutions in ZmGA20ox3, focusing in particular on the 25 mutations catalogued in the database (Fig. 3c). Notably, the negative effects of G293D and L262F rank within the top 10% of all possible mutations, consistent with our objective of attenuating GA biosynthesis and reducing plant height. Both mutations are close to the GA53-binding pocket, and their substitutions lead to conformational changes of GA53 within the pocket, accompanied by alterations in a series of bonds involved in GA53 binding (Fig. 3d). These mutants merit field trials to assess phenotypic effects. If reduced plant height occurs in the B73 inbred line, markers for these causal mutations could support molecular breeding.

**Figure 4:**
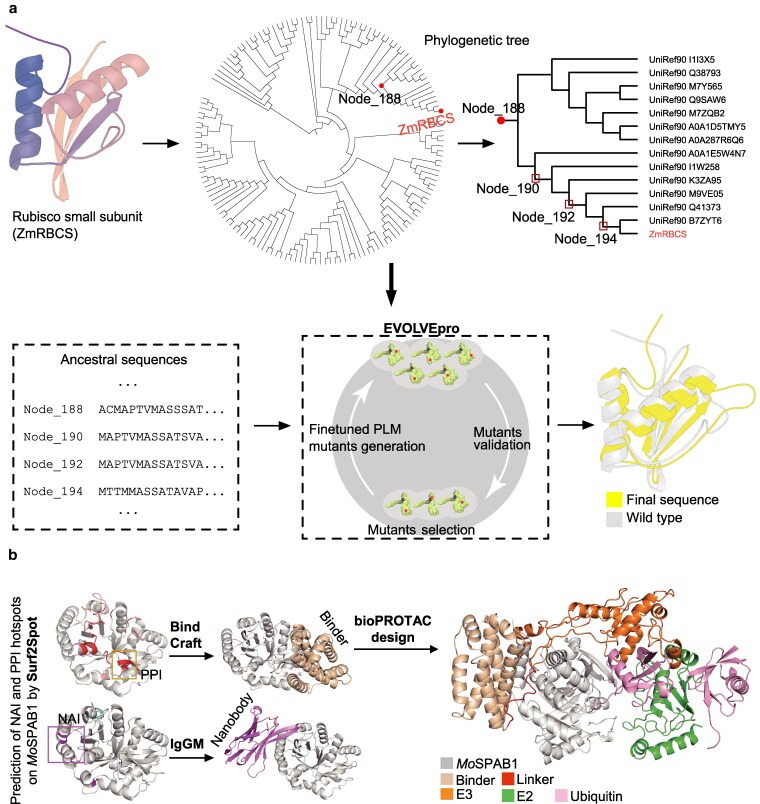
Refactoring and de novo design for plant breeding. **(a)** Schematic illustration of maize Rubisco small subunit (Zm00001eb197410, ZmRBCS) redesign via ancestral sequence reconstruction. Homologous sequences at phylogenetic branch nodes are identified using FireProt ASR to reconstruct ancestral RbcS sequences. These sequences fine-tune the protein language model in EVOLVOpro to enhance ME prediction. Experimentally screened top-performing ancestral sequences serve as starting points for iterative optimization, introducing beneficial mutations to improve thermal stability and enzymatic activity. **(b)** The secreted protein MoSPAB1 from Magnaporthe oryzae as a target for nanobody and bioPROTAC design. Hotspot residues at nanobody–antigen interaction (NAI) and protein–protein interaction (PPI) sites are predicted by Surf2Spot. Binders and nanobodies targeting these sites are designed by BindCraft and IgGM, respectively, with predicted hotspots as anchors. Complex conformations can be simulated to identify optimal binders and linkers for bioPROTAC construction. The related predictive structures are generated by AF3.

Refactoring design can improve stability and activity of plant proteins. Using the maize Rubisco small subunit as an example, we can collect homologous sequences, reconstruct ancestral variants with FireProt ASR (Khan et al. 2021), and feed them into EVOLVEpro to enhance ME prediction (Matthews et al. 2024). Experimentally validated variants can then undergo iterative optimization to yield Rubisco subunits with greater thermal stability and photosynthetic activity (Fig. 4a). De novo design enables entirely new traits by creating proteins absent in nature. Development of inhibitory binders against the SARS-CoV-2 spike protein illustrates this potential (Cao et al. 2020; Case et al. 2021; Pomplun et al. 2021; Hunt et al. 2022). Similar strategies could target plant pathogens, with transgenic expression of de novo binders or engineered bioPROTACs neutralizing virulence factors. BioPROTACs mimic E3–E2∼Ub complexes, directing proteasomal degradation of specific proteins, and thus represent synthetic traits. For example, broad-spectrum bioPROTACs could be designed to inhibit fungal effectors from *Magnaporthe oryzae*, providing rice with durable resistance (Fig. 4b).

## Conclusions and path forward

AI is accelerating the adoption of PLMs and their derivative prediction and design tools across the life sciences, with the potential to profoundly transform plant research and breeding. In this review, we have outlined the principles, models, and tools of AI-enabled protein design and structural biology, and discussed their implications for plant science and crop improvement. Through hypothetical scenarios, we illustrate how the AI4S paradigm can reshape plant research by shifting it from observation-driven approaches toward model-driven, AI-powered discovery, and engineering.

Plant systems offer several unique advantages for AI-driven protein design, including efficient in planta expression, low immunogenicity, cost-effective functional validation, and the ability to directly select improved crop lines (Li et al. 2025). Nevertheless, substantial challenges remain (see Outstanding Questions), particularly due to the limited availability of experimentally resolved structural data and the inherent complexity of plant biological systems (Armstrong et al. 2023; Haley et al. 2025; Lin et al. 2025). First, the scarcity of structural, functional, and molecular interaction data in plants, coupled with the high diversity of plant proteins and species, constrains the performance of PLMs and other DL tools. A critical challenge is therefore how to exploit fundamental principles, such as physicochemical representation and transfer learning, to develop models more specifically tailored to plant biology. Second, the practical application of protein design currently requires substantial expertise in both AI and programming. A major next step is the development of integrated AI4S agents that combine PLM-based predictions with established design tools in user-friendly platforms, thereby streamlining workflows and providing interpretable outputs accessible to plant scientists. Third, the creation of AI agents specifically oriented toward breeding applications is essential. Such systems will necessarily be more complex than those designed for basic research, as they must connect upstream computational design with downstream biotechnological implementation and integrate molecular-level data with organism-level phenotypic information to support AI-assisted breeding decisions. Fourth, the expression of synthetic proteins in plants may introduce unforeseen biosafety risks to ecosystems and human health. Robust regulatory frameworks and risk assessment strategies are therefore required, and approaches to mitigate potential risks during the design stage via model-based prediction should be actively explored. Finally, the scope of protein design extends beyond agronomic trait improvement. Its applications in plant-based synthetic biology and bio-manufacturing, such as the design of bio-nanomaterials, biosensors, antimicrobial peptides, and therapeutic proteins, warrant further investigation to expand plant functionalities.

Although AI-driven bio-breeding remains at a proof-of-concept stage, its continued development is expected to catalyze a paradigm shift in plant breeding, moving from “discovering and understanding traits” toward “designing and synthesizing traits.” Importantly, protein design does not operate in isolation. Its successful deployment relies on integration with high-throughput experimental screening for functional validation, genome editing technologies for precise genetic modification, and extensive field trials to assess the performance, stability, and adaptability of artificial variants or engineered proteins in crop plants.

## Advances box

- AI is accelerating the adoption of PLMs and their derivative prediction and design tools across the life sciences, poised to transform plant research and breeding.
- General principles and widely applicable models for protein understanding and generation have been established, with case studies demonstrating their utility in advancing plant research and breeding.
- Structure-aware interpretations of mutation–function relationships enable precise hypothesis generation and support experimental validation through protein design tools.
- DL-driven protein engineering, including rational, semi-rational, refactoring, and de novo design, creates artificial variants and synthetic proteins with enhanced or novel functions to accelerate plant breeding.

## Outstanding questions box

- How can physicochemical principles and transfer learning be used to develop protein language models tailored to plant diverse structural and molecular data gaps?
- What strategies can create AI4S agents that combine protein language model predictions with design tools into user-friendly platforms for non-expert protein design?
- How can AI-driven bio-breeding platforms integrate computational protein design with biotechnological tools, linking molecular data and phenotypes for effective breeding decisions?
- What regulatory frameworks and risk assessments are needed for biosafety of synthetic proteins in plants, and how can model-based design mitigate risks early?
- How can protein design extend beyond agronomic traits to advance synthetic biology and bio-manufacturing applications like bio-nanomaterials, biosensors, antimicrobial peptides, and therapeutics?

## Supplementary Material

kiag147_Supplementary_Data

## Data Availability

No new data were generated or analysed in support of this research.
