# Supplementary material for: AI-enabled protein design facilitates future plant research and crop breeding
Source: Plant Physiol. 2026 Mar 18;200(4):kiag147. doi: 10.1093/plphys/kiag147 (PMC13048276; doi:10.1093/plphys/kiag147)
Supplement: kiag147_Supplementary_Data [file kiag147_supplementary_data.pdf]

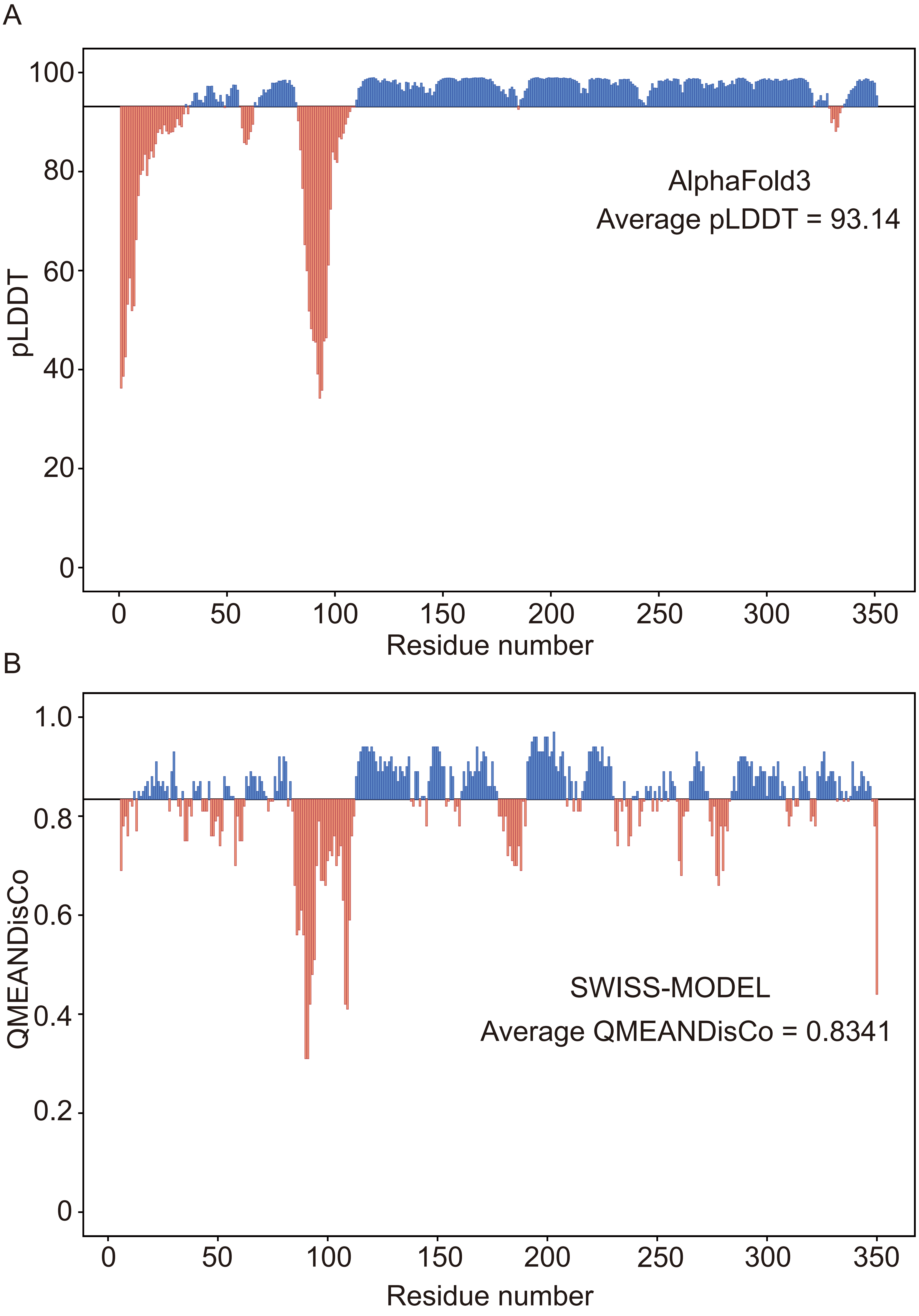
A
100
80
60
40
20
0
pLDDT
AlphaFold3
Average pLDDT = 93.14
0
50
100
150
200
250
300
350
Residue number
B
1.0
0.8
0.6
0.4
0.2
0
QMEANDisCo
SWISS-MODEL
Average QMEANDisCo = 0.8341
0
50
100
150
200
250
300
350
Residue number

Figure S1. Confidence assessment of structural models predicted by AF3 and SWISS-MODEL. A) Per-residue pLDDT scores of the ZmGID1 model predicted by AF3. B) Per-residue QMEANDisCo scores of the ZmGID1 model predicted by SWISS-MODEL, which was generated using the crystal structure of Arabidopsis GID1 (chain A of PDB ID 2ZSI) as the homologous template.

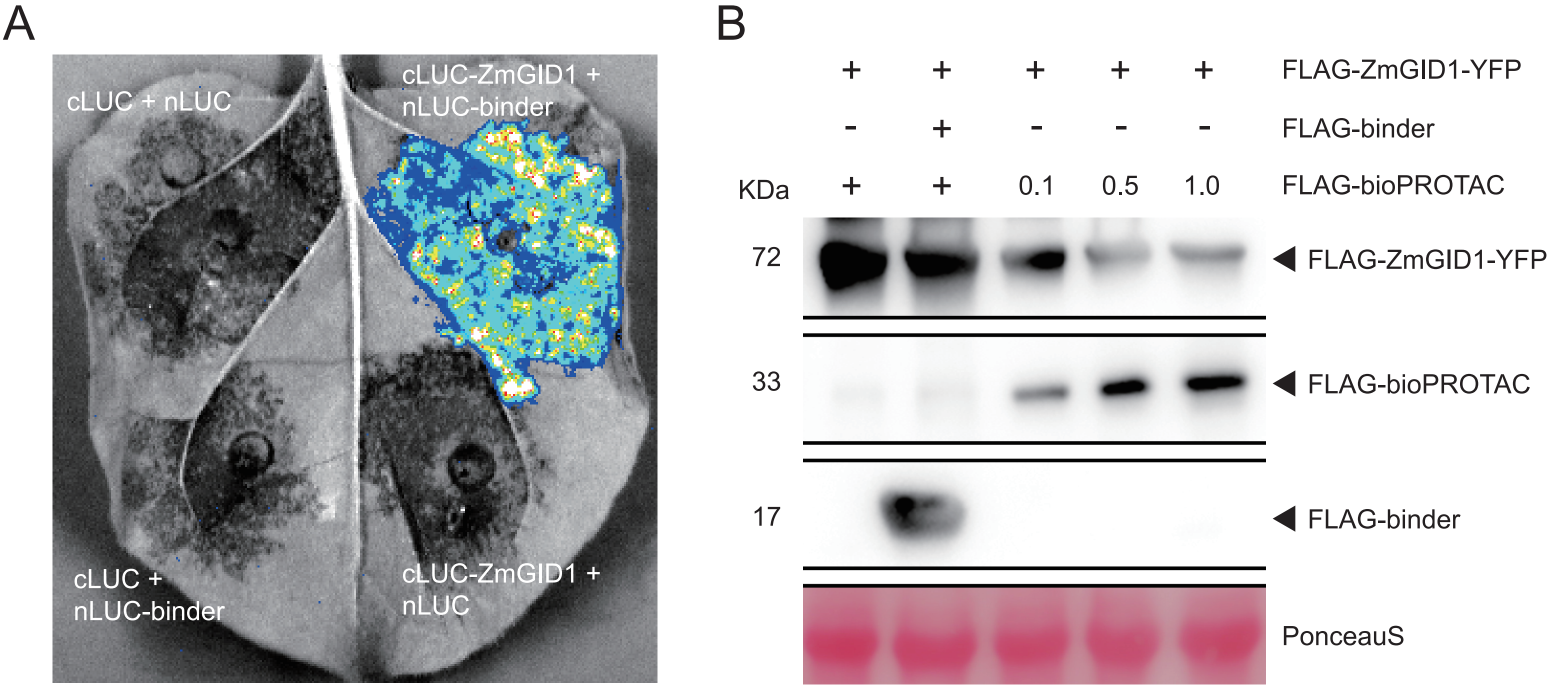

Figure S2. Validation of binder interaction and target protein degradation. A) LUC assay to detect the physical interaction between the binder and ZmGID1. Fusion constructs of cLUC-ZmGID1 and nLUC-binder were transiently co-expressed in Nicotiana benthamiana leaves. Negative controls included cLUC + nLUC-binder, cLUC-ZmGID1 + nLUC, and empty vector combinations. At 48 hours post-infiltration, D-luciferin substrate was infiltrated, and luminescence signals were recorded using a chemiluminescence imaging system. A strong luminescence signal was specifically detected in the cLUC-ZmGID1 + nLUC-binder combination compared with all controls, indicating a specific interaction between the binder and ZmGID1. B) Western blot analysis of bioPROTAC-mediated degradation of the target protein. FLAG-tagged bioPROTAC and ZmGID1-YFP fusion proteins were co-expressed in tobacco leaves with increasing amounts of bioPROTAC plasmid (0.1, 0.5, 1.0 μg). Total protein was extracted at 72 hours post-infiltration. Anti-FLAG antibody was used to verify bioPROTAC expression and to detect ZmGID1-YFP protein levels. The results show a dose-dependent reduction in ZmGID1-YFP abundance with increased bioPROTAC expression, confirming effective target degradation. Ponceau S staining was used as a loading control.
